# The Potential for Zinc Stable Isotope Techniques and Modelling to Determine Optimal Zinc Supplementation

**DOI:** 10.3390/nu7064271

**Published:** 2015-05-29

**Authors:** Cuong D. Tran, Geetha L. Gopalsamy, Elissa K. Mortimer, Graeme P. Young

**Affiliations:** 1 CSIRO Food and Nutrition Flagship, Gate 13, Kintore Ave, Adelaide SA 5000, Australia; E-Mail: geetha.gopalsamy@flinders.edu.au; 2 School of Medical Sciences, Faculty of Health Sciences, The University of Adelaide, Adelaide SA 5005, Australia; 3 Flinders University of South Australia, Bedford Park, GPO Box 2100, Adelaide SA 5001, Australia; E-Mails: elissa.mortimer@flinders.edu.au (E.K.M.); graeme.young@flinders.edu.au (G.P.Y.)

**Keywords:** zinc, diarrhoea, kinetics, stable isotope, modelling, child health, global health

## Abstract

It is well recognised that zinc deficiency is a major global public health issue, particularly in young children in low-income countries with diarrhoea and environmental enteropathy. Zinc supplementation is regarded as a powerful tool to correct zinc deficiency as well as to treat a variety of physiologic and pathologic conditions. However, the dose and frequency of its use as well as the choice of zinc salt are not clearly defined regardless of whether it is used to treat a disease or correct a nutritional deficiency. We discuss the application of zinc stable isotope tracer techniques to assess zinc physiology, metabolism and homeostasis and how these can address knowledge gaps in zinc supplementation pharmacokinetics. This may help to resolve optimal dose, frequency, length of administration, timing of delivery to food intake and choice of zinc compound. It appears that long-term preventive supplementation can be administered much less frequently than daily but more research needs to be undertaken to better understand how best to intervene with zinc in children at risk of zinc deficiency. Stable isotope techniques, linked with saturation response and compartmental modelling, also have the potential to assist in the continued search for simple markers of zinc status in health, malnutrition and disease.

## 1. Introduction

Zinc is recognised as an essential micronutrient for normal growth and development as well as for achieving and maintaining health. Accordingly, there has been a great expansion in the recognition of the clinical and public health significance of this important micronutrient [1]. The application of zinc as a drug to treat diseases is also increasing due to advances in our understanding of the diversity, versatility and extraordinary importance of zinc in cellular biology [2]. Micronutrient deficiency affects more than 50% of the world’s population, particularly in low-middle income countries [3]. It is estimated that zinc deficiency affects about 1.2 billion people worldwide [3]. The health consequences of zinc deficiency include impairments in growth, intellectual development and reproductive health [4]. Infants, children and women of child-bearing age are particularly vulnerable. Epidemiological studies have shown that zinc deficiency increases the risk of diarrhoea in young children by 33% [5], that of pneumonia by 69% and that of malaria by 56% [6].

The beneficial effects of zinc administration include reduction in the incidence of diarrhoea and pneumonia [1,7], and the rate of mortality [8,9,10,11,12,13,14] among young children in low-middle income countries. A review [9] showed that providing zinc supplements reduced overall child mortality by 6% in deficient populations and reduced deaths of children over 1 year of age by 18%. Zinc supplementation has also been demonstrated to increase the growth of stunted children [15]. However, the optimal dose of zinc required to achieve such beneficial outcomes, while avoiding potential adverse effects of excessive zinc intake, remains largely unknown. 

Recommendations on how much zinc to give are confusing. Previously, the recommended dietary allowance (RDA) of zinc for preschool children was set at 10 mg/d by national and international organisations [16,17]. However, the US Food and Nutrition Board (Institute of Medicine), the International Zinc Nutritional Consultative Group (IZiNCG), and the World Health Organisation (WHO) now suggest a lower RDA of 3 mg zinc/day for 1–3 year old children [18]. In the 2008 Copenhagen Consensus statement [19], zinc plus vitamin A supplementation was the highest ranked solution for advancing global wellbeing and health. Despite this endorsement of zinc supplementation for children under the age of 5 years, dosage or scheduling frequency was not stipulated. Currently, the only global directive regarding zinc supplementation is that recommended in the 2004 WHO/UNICEF Joint Statement on management of Acute Diarrhoea which recommends 10–20 mg of zinc for 10–14 days in the case of an acute episode of diarrhoea [20]. 

Despite these recommendations, zinc administration has not yet been widely adopted [21,22]. While a range of factors contributes to this [22,23], uncertainty about dose, scheduling and context of use are contributory factors. 

The purpose of this paper is to review our current understanding of human zinc physiology and homeostasis, with a specific focus on tracer studies using zinc stable isotopes and compartmental modelling techniques. We also discuss the evidence from human studies to address knowledge gaps on the pharmacokinetics of zinc administration, specifically in relation to optimal dose and scheduling where gut function is compromised. Finally, we discuss how stable isotope techniques, linked with saturation response and compartmental modelling, have the potential to assist in the continued search for simple markers of zinc status in health, malnutrition and disease.

## 2. Techniques for Measuring Zinc Physiology 

Radio-tracer techniques have made important contributions to the knowledge of human zinc physiology over a period of 50–60 years. Prior to the 1980s, tracer studies were used to develop detailed compartmental models of zinc metabolism [24] and to study zinc absorption and bioavailability [25]. Some of the first zinc kinetics studies used radioactive labelled zinc (^65^Zn), which allows measurement of intestinal zinc transport without the interference of the presence of endogenous zinc [26,27]. The disadvantage of using radio-labelled zinc is the extraordinarily long radioactive and biological half-life of ^65^Zn. Early studies [28,29] showed that radioactive label still remained in the body 14 days after administration. However, there are also advantages to using ^65^Zn tracers since zinc absorption, distribution and excretion and the quantity of zinc in organs such as liver, muscle and bones and the whole body can be observed for up to 9 months after the dose is administered [27].

Our understanding of whole body zinc homeostasis and physiology has been greatly enhanced by the application of zinc stable isotope tracer techniques and advances in analytical instrumentation, especially in the development and application of inductively coupled plasma mass spectrometry (ICP-MS) [30,31,32,33]. For instance, dual stable isotope tracer techniques to measure fractional absorption of zinc (FAZ) require only a single measurement in plasma or urine of both orally and intravenously administered zinc tracers [32]. In addition, methodologies such as faecal monitoring, deconvolution and indicator dilution have also been developed to assess zinc homeostasis [34]. 

The dual zinc stable isotope tracer techniques have been used to quantify the primary parameters that assess zinc homeostasis [35] namely FAZ and thereby total absorbed zinc (TAZ), endogenous faecal zinc (EFZ), exchangeable zinc pool (EZP) and net zinc retention (NZR) [35,36,37,38,39,40,41]. Multi-tracer techniques utilising kinetic data derived from administration of different zinc stable isotopes intravenously and/or orally under different conditions can be used for compartmental modelling of zinc metabolism. Another application of this method can be used to compare the effects of different diets and different chemical forms of zinc on zinc bioavailability and homeostasis [41].

Zinc stable isotope techniques have several advantages over radioisotope techniques: They are non-radioactive and can therefore be used in studies on women of child bearing age and young infants. Another advantage is that multiple zinc tracers can be administered to any one study subject, enabling independent monitoring of the kinetics of these various tracers [39]. 

## 3. Assessment of Zinc Physiology Using Zinc Stable Isotopes

### 3.1. Intestinal Regulation of Zinc Homeostasis

The small intestine is considered to play a major role in zinc metabolism and homeostasis. It has been generally accepted that zinc homeostasis is achieved by absorption of exogenous dietary zinc and conservation of endogenous zinc lost into the intestine [42]. The former is subject to dietary zinc content and bioavailability while the latter is affected by gut disease and enteropathy [42,43]. Homeostatic regulation is crucial since excess zinc can lead to cytotoxicity [44]. In this section, we will describe how zinc stable isotopes have been used to explore the regulation of zinc absorption and endogenous intestinal secretion/excretion in response to changes in dietary zinc. Concomitant with this discussion is the concept of zinc bioavailability and the important inhibitory role which dietary phytic acid may have on zinc absorption. 

### 3.2. Zinc Bioavailability and Phytic Acid

Zinc is generally bound/complexed by organic substances like proteins, amino acids, organic acids and other ligands facilitating higher bioavailability than when bound to inorganic compounds [45]. This may be due to a higher solubility of these complexes as well as to the existence of specific intestinal absorption mechanisms for organic ligands which transfer complexed compounds into the enterocytes [46,47]. The three main dietary factors influencing absorption and ultilisation of zinc are phytic acid [28,48], the total zinc content of the meal [26,28] and the amount and source of protein [26,29,49]. Of these, the most critical factor affecting the bioavailability of zinc is the phytic acid content of the diet. Phytate or phytic acid is a main storage form of phosphate and is ubiquitously distributed in plant foods, especially cereal grains and legumes. Phytate restricts zinc and other mineral bioavailability by forming insoluble complexes [50]. High dietary phytate is regarded as the major reason for widespread zinc deficiency in the developing world. Strategies to reduce dietary phytate content include soaking, fermentation, malting and germination, which have been used successfully in various locations [51,52,53,54]. 

### 3.3. Measurement of Zinc Absorption

Stable isotope tracer methods have now been used in both children and adults for the assessment of zinc absorption [55,56,57,58]. In the 1990s, Friel *et al.* [32] developed a new method for measuring FAZ. Only a fraction of the oral zinc dose is enriched with the tracer, and a different zinc stable isotope is injected intravenously. Urine monitoring possibly with multiple venepunctures and/or long faecal collection periods are needed depending on the endpoints measured [32]. 

Analysis of two stable isotopes in urine permits calculation of FAZ; the following equation is used [59]:

**FAZ(urine; %) = enrichment (oral/intravenous) × dose (intravenous/oral)**

Assumptions:

(i) That both isotopes (oral and intravenous) enter the plasma at the same time.
(ii) That the isotope enrichment in plasma and urine are the same after 40 hrs (enough time has elapsed since isotope administration so that the intravenous and oral tracer enrichments are decaying proportionally).
(iii) That urine enrichments of both isotopes are similar for plasma.
(iv) That the extrinsic label isotope reflects that of the intrinsic zinc in the diet.

FAZ studies have shown that the amount of zinc in a meal will affect zinc absorption, *i.e.*, with increasing levels of zinc in a meal, FAZ will decrease. Not surprisingly, early studies [31,60,61,62] have shown that reducing dietary zinc increases FAZ [60,62,63], whereas doubling the zinc levels caused FAZ to decline, thus confirming an inverse relationship between the quantity of zinc ingested and the FAZ [64,65,66]. The decline in fractional absorption with increasing dietary zinc is an important factor in maintaining zinc homeostasis when intake is excessive [27]. 

However, it is the TAZ per day, rather than fractional absorption that seems most relevant to actual zinc replacement. For the determination of TAZ, the following equation is used [59]:

**TAZ (mg/day) = FAZ ×Total dietary zinc intake (mg/day)**

Assumptions: as for FAZ.

At low dietary zinc intakes, TAZ progressively declines directly with the severity of zinc restriction [64]. Although there is a gradual increase in FAZ at low zinc intakes, this progressive increase in fractional absorption of the available zinc appears to be inadequate to maintain zinc homeostasis [67]. 

### 3.4. Measurement of Endogenous Faecal Zinc (EFZ) Excretion 

EFZ is defined as the quantity of endogenous zinc excreted in the faeces, representing the difference between that being secreted into the lumen and that which is reabsorbed. EFZ is considered fundamental to the conservation of zinc [68]. The gut is the major route of zinc excretion by the body; at least twice as much is excreted in faeces compared to all other routes including desquamation [42,60]. The quantity of endogenous zinc excreted via the intestine depends on both recent [63,69] and long term [60,67] zinc intake over a wide range of ingested zinc [70]. EFZ is not a constant but typically varies with TAZ. Caution is required when comparing EFZ [36]. Temporary increases in FAZ have been observed when dietary zinc is reduced, however increases in FAZ are not maintained [60]. Populations with habitually low dietary zinc intakes have correspondingly low EFZ [67]. It has been demonstrated that low absorption of exogenous dietary zinc is associated with low endogenous losses in the faeces [60,67] and low readily exchangeable body zinc pools [67]. These findings suggest that excretion of endogenous intestinal zinc is considered a major mechanism for conserving intestinal zinc, enabling the restoration or maintenance of zinc balance when zinc intake is reduced or low [39]. The overall conservation in EFZ excretion might reflect a reduction in the amount of zinc secreted into the gut as well as an increased re-absorption of the endogenous zinc due to the up-regulation of specific zinc transporters [71,72].

Zinc may be lost into the lumen via either physiological losses from cell shedding and intestinal secretions which contain zinc-proteins [70] or pathological losses due to impaired gut function and disease [73]. Failure to conserve EFZ may be a primary cause of zinc deficiency and is common in in young children with diarrhoea [5,74,75]. In response to the excess loss of endogenous zinc, homeostasis is maintained by increasing the retention of absorbed zinc [60,67]. EFZ loss can be estimated from the amount of zinc excreted in the stool (using stable isotope methods) when a zinc-free diet is fed and is estimated to be approximately 6–8 μmol/day [76]. Adjustments in excretion of intestinal endogenous zinc to changes in zinc intake can be maintained over prolonged periods [60,67].

EFZ can be measured using the stable isotope tracer technique described by Jackson *et al.* [77] and Turnlund *et al.* [78]. Furthermore, EFZ can also be measured using compartmental models [79,80], however, this requires complete urine and faecal collections and the measurement of tracer excretion in both [38,56]. As noted by Miller *et al.* [80], compartmental models of biological systems are a simplistic approximation of very complex systems. Caution is therefore needed in the interpretation and extrapolation of compartmental models. Furthermore, it is important to be aware that modelling is “a working hypothesis and the best objective and subjective integration of the current state of knowledge” [81] and that it provides important measures of zinc metabolism not directly measurable [79]. Lowe *et al.* [79] also reported that not all body zinc was apparent from the modelling of isotope data acquired. For the determination of EFZ, the following equation is used [36]:

**EFZ (mg) = Σ (F × f)/(u × t)**

**F** is total faecal Zn during the metabolic period (mg); **f** is the corresponding faecal percent enrichment (%E) stable isotope Zn; **u** is the mean urine %E stable isotope Zn during the metabolic period; and **t** is time of the metabolic period (day).

Assumptions:

(i) That the endogenous zinc excreted in the faeces is derived from a pool of zinc that exchanges rapidly with zinc in plasma and in certain solid tissues.
(ii) That there will be some unabsorbed label in the faeces compared to label that has been absorbed and then secreted into the intestine and excreted with the faeces.
(iii) That there will be no unabsorbed quantity of the same tracer used to label the urine in the faeces during the metabolic period.

### 3.5. Assessment of Readily Exchangeable Zinc Pools (EZP)

Another important factor in zinc metabolism and homeostasis that needs consideration is the size of the ‘pools’ of zinc within the body with which plasma zinc readily exchanges. This concept of the ‘rapidly’ EZP was first derived from radioisotope studies combined with model-based compartmental analysis, which identified several compartments that exchange completely with plasma within a 2-day period [27]. These studies found that EZP accounts for only approximately 10% of the total body zinc content but seems to have a vital role in a wide range of zinc-dependent metabolic processes [82]. 

Subsequently, Miller *et al.* [83] have used zinc stable isotope tracer techniques to assess the EZP, and they have provided further insight into zinc metabolism, homeostasis and status [67,80,83]. Measurements of the size of EZP have demonstrated a positive correlation with dietary zinc intake, the quantities of zinc absorbed each day and with daily excretion of endogenous faecal zinc [67]. Estimates of EZP size can be obtained from plasma- or urine-enrichment data after intravenous administration of a zinc stable isotope tracer [83]. Estimates of EZP size can also be derived from urine enrichment after oral administration of a zinc tracer, provided there is a simultaneous measurement of FAZ [55]. The latter is particularly useful for applications in field studies of young infants where intravenous administration is often not feasible. For the determination of EZP, the following equation is used [59]:

**EZP (mg) = intravenous dose (mg)/y intercept**

The y intercept was estimated by extrapolation from a linear regression analysis of the natural log of the percentage enrichment of the intravenous isotope in the five daily urine samples. Sampling should take place 2–3 days after tracer administration to give the tracer adequate time to distribute throughout the EZP.

Assumptions:

(i) That the EZP accounting for 10% of total body zinc in adults is considered to be particularly important for zinc-dependent biological processes.
(ii) That estimates of EZP size can be obtained from plasma or urine enrichment data after intravenous administration of a zinc stable isotope tracer.

Reduction in EZP size has been demonstrated during periods of marginal zinc intake [67] and the presence of impaired gut function such as celiac disease [58]. The intestine only conserves endogenous zinc when the EZP is depleted [67]. These findings suggest that zinc restriction and gut inflammation, which often occur in low-middle income countries, may affect the size of the EZP and hence available body ‘stores’. 

### 3.6. Limitations of Zinc Stable Isotope Techniques

Historically, there have been difficulties inherent in conducting isotope studies: purchasing high-quality mineral stable isotopes has been problematic [39] as has the analysis of biological samples [84]. Some major limitations with the use of zinc stable isotope techniques include the relative expense of the required analytical instrumentation, such as state-of-the art ICP-MS and the high level of laboratory expertise needed to obtain reliable measurements of stable-isotope ratios [39]. Therefore, only a limited number of laboratories, principally in the United States and Europe are able to analyze the samples using mass spectrometry. The technique is somewhat invasive, requiring a single venepuncture to place an intravenous catheter for administration of intravenous zinc.

## 4. Utilisation of Zinc Stable Isotope to Model Zinc Kinetics

### 4.1. Saturation Response Kinetic Modelling

Condomina *et al.* [85] considered that the Michaelis-Menten kinetic model was the best for describing the transport profile of zinc through the small intestine. This model predicted bioavailable zinc intakes and dietary factors and their interactions influencing zinc absorption [86]. 

**Figure 1:**
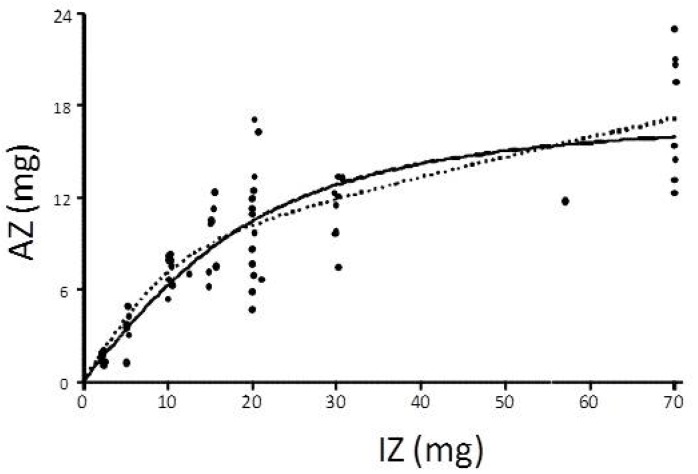
The individual measurements of absorbed Zn (AZ) vs ingested Zn (IZ), one time doses given in the post-absorptive state, and the fitted Hill Equation model (thick solid line). The model predicts the absorption, approaching the A_max_ value of 13 mg (dashed line). The Hill equation model is represented by the following equation; $\mathrm{AZ}=\frac{A_{\max}*{\mathrm{IZ}}^{H}}{{\mathrm{IZ}}^{H}+{\mathrm{LA}}_{\mathrm{50}}^{H}}$ where the parameters *A_max_*, *IA_50_*, and *H* are the maximum absorbed Zn, the ingested Zn (IZ) that results in absorbed Zn (AZ) of 50% of *A_max_*, and the Hill (or sigmoidicity) coefficient, respectively.

Using this approach, Tran *et al.* [87] have shown that the approximate saturation point was reached when intake of zinc reached 20 mg/day. This dose resulted in a TAZ of 11 mg when administered to healthy adults as an aqueous solution postprandially with little being achieved by administering greater amounts (Figure 1). 

Istfan *et al.* [88] suggested that there was a hyperbolic relationship between ingested zinc and FAZ with an asymptote for zinc absorption of 56%. However, Tran *et al.* [87] showed that FAZ fell when ingested zinc was above 24.5 mg. Although FAZ decreases with increasing Zn ingested, the actual amount of zinc absorbed continues to increase, albeit at a slower rate. These observations are consistent with an active transport mechanism, which becomes saturated at about 20 mg/day, with only passive diffusion accounting for incremental absorption achieved by doses above this (Figure 1). 

It is now well accepted to use the saturable response model for zinc absorption. Saturation kinetics analyses of quantitative data on daily zinc absorption *versus* intake provide a biologically relevant means of modelling and interpreting experimental data. Important parameters to be determined in zinc kinetic studies are; K_m_; the half saturation constant (Michaelis constant), V_max_; the maximum transport rate, K_d_; the passive permeation coefficient. The optimal dose is hypothesized to approximate that which saturates the active transport mechanisms, based on the one phase exponential association regression, V_max_, which is the maximal binding/transport and is reached when ingested Zn is approximated to 100 mg.

Application of the saturation response modelling used to calculate TAZ:

**TAZ = (A_max_ × TDZ)/(IA_50_ × TDZ)**

**A_max_** (equates to **V_max_**) is the maximal absorption of zinc; and **IA_50_** (equates to **K_m_**) is the quantity of zinc intake required for half maximal absorption [59].

Assumptions:

(i) That the saturation response model fits a Michaelis-Menten kinetics curve

Although sufficient data have been accumulated for adults and infants for saturation kinetic modelling for zinc absorption, it should be recognized that optimal data are not yet available. This is due to the fact that saturation kinetic modelling is based on mean absorption data from studies in humans conducted by multiple researchers. Currently, there is no single study that has adequately addressed the saturation kinetic modelling (zinc dose response) to characterise quantitative changes in zinc absorption with various concentrations of ingested zinc in children. 

### 4.2. Compartmental Modelling

Besides the saturation kinetic modelling, there is another type of zinc kinetic modelling, which involves model-based compartmental analysis. Compartmental modelling is becoming increasingly important in exploring and advancing the understanding of the complexities of mammalian whole body zinc metabolism and homeostasis [89].

Several compartmental models of zinc metabolism and homeostasis have been reported [27,79,80,90,91,92,93] and the complexity of each varies according to the amount of data to be fitted and the number of physiologic compartments included. Compartmental analyses have identified that the liver plays a central role in zinc metabolism in that it is part of the EZP [27], together with other rapidly exchanging compartments such as the kidney and spleen [94] and bone marrow [89]. Compartmental model analysis has allowed the evaluation of estimates of the quantity of the EZP with considerable accuracy [67,80,83]. 

A number of investigators have used mathematical functions and regression analysis to model the observed relations between zinc absorption and various dietary components, including zinc and phytate [3,18,49,50,59,86,87,95,96,97,98]. Miller *et al.* [99] have established a new mathematical modelling method for zinc absorption as a function of dietary zinc and phytate. This proposed model is unique in that it is based on a biochemical conception of the absorption process, was tested based on its fit to selected data from the literature [99] and was developed as a continuation of the application of the saturation response modelling (derived from pharmacokinetics data analysis) to zinc absorption data [59,87,98,100]. Miller *et al.* [99] concluded that the mathematical model of zinc absorption is a valid model with immediate relevance and applicability to the study of zinc nutrition and metabolism and the estimation of dietary zinc requirements. 

## 5. Implications for Zinc Supplementation

In 2004, WHO and UNICEF took two significant steps to reduce diarrheal diseases in children by recommending the use of oral rehydration solution (ORS) and supplementation with zinc for up to two weeks following an acute attack of diarrhoea [20]. The zinc recommendation was based on the results of several randomized controlled trials, meta-analyses [101] and reviews [16,17,102]. 

Further evidence for the use of zinc supplementation over longer periods is summarised in a series of meta-analyses [9,22,103] where supplementation duration ranged from 2 weeks [104] to 15 months [105] and the dose ranged from 1 to 70 mg/day. In addition to dose and duration, there was no consistency in chemical forms of zinc used, as pointed out by a recent systematic review [23]. 

The following discussion addresses what is known from dual isotope tracer studies that might inform the appropriate zinc dosing regimen.

### 5.1. Zinc Dose and Amount Absorbed

Most studies conducted into zinc supplementation as a treatment for disease have used varying zinc solutions, doses and frequencies of administration. There are limited data available regarding dose-response, and what is available is from short-term relatively high-dose supplementation [106,107]. Most studies regarding zinc supplementation to improve immune function or growth and development of children have used between 10 and 20 mg of zinc/day [108,109,110]. 

Very few studies have used clinical outcomes to assess dose response. Wuehler *et al.* [111] tested 3, 7 or 10 mg zinc/day in Ecuadorian children at risk of zinc deficiency and showed that zinc supplementation with a dose as low as 3 mg/d for 6 months increased plasma zinc concentrations and reduced incidence of diarrhoea. No observed adverse effects were seen at 10 mg zinc/day. By contrast, in an open-label randomised clinical trial [112] conducted in well-nourished Turkish children, zinc therapy (15–30 mg/day) increased plasma zinc levels, but did not change either the duration or severity of the diarrhoea. No long-term studies using zinc stable isotope techniques have been undertaken in such settings, so we do not know if these very different responses depend on differences in the amount of zinc absorbed or conserved or if the size of the EZP is important. The use of zinc stable isotope techniques in such studies would greatly inform the interpretation of these results.

Tran *et al.* [87] were the first to undertake a zinc stable isotope study to examine the relationship between the dose of zinc given in the post-absorptive state and the amount absorbed. In normal adults [87], they found that for zinc sulphate, single doses above 20 mg resulted in small and progressively diminishing increases in TAZ, suggesting dosages above this amount provided a diminishing return. No such data are available for children, especially those in susceptible populations. Tracer studies in adults and children have shown that the total amount of absorbed zinc increases linearly in relation to the test dose, with doses ranging from 1 to 6 mg zinc. However, the magnitude of the increase in zinc absorption is progressively less with higher doses, *i.e.*, 9–30 mg zinc/day [66,87].

### 5.2. Frequency and Duration of Administration?

There is considerable variation in both the frequency and duration of zinc supplementation for the prevention of diarrhoea in children [22,23]. The duration of supplementation ranges from weeks to months and even years [22], and frequency of supplementation can be daily, intermittent or weekly [23] when given to children. WHO and UNICEF recommend a 10- to 14-day course of zinc treatment in addition to ORS for the treatment of acute childhood diarrhoea [20]. In none of these situations is the dose informed by kinetic studies of zinc administration but rather by perceived daily requirements, considering what might be both tolerated and safe. 

***Short-term supplementation***—Sandstrom *et al.* [113] and Sandstrom and Cederblad [26] were first to recognise that there was an inverse relationship between the amount of ingested zinc and FAZ during short-term zinc supplementation. Istfan *et al.* [88], reported consistent absorption between days 2 and 10 of consuming a formula diet with 15 mg zinc/day and, after the diet was reduced to <2 mg zinc/day on day 12, a consistently greater absorption on days 18 and 28. Furthermore, Hunt *et al.* [114] demonstrated that the absorptive capacity was greatest if dietary zinc intake was below 11 mg/day, corresponding to an absorption of <4.1 mg/day. 

***Long-term supplementation***—The short-term effect of zinc supplementation has confounded the interpretation of longer-term observations of increased FAZ with zinc depletion [60,62,63,115]. For instance, an increase in FAZ from 25% of 16.4 mg dietary zinc to 49%–53% after 13–42 day of 5.5 mg dietary zinc, was reported by Wada *et al.* [62], consistent with Hunt *et al.* [114]. These findings showed that humans adaptively increase FAZ, beyond the immediate and substantial influence of the ingested zinc dose, when consuming low zinc diets. The observed adaptation in zinc absorption from the low zinc, higher bioavailability diets appears to occur within 4 weeks [114]. This observation is consistent with the report of Lee *et al.* [60] that men’s zinc absorption does not change between 2, 4 and 6 months of consuming 4.1 mg dietary zinc.

In a recent systematic review and meta-analysis [22] it was reported that the trials supplementing zinc for a duration of 10–26 weeks [109,116,117,118,119,120,121] showed a reduced likelihood of diarrhoea but trials using zinc supplementation for shorter [10,104,122] or longer [14,105,123,124,125,126,127] duration than this interval did not show a benefit for the outcome of diarrheal incidence. These inconsistencies might relate to differing dosing frequencies, and no published data serve to guide one as to how often zinc should be given to achieve maximum absorption, and whether zinc status has any influence. 

**Figure 2:**
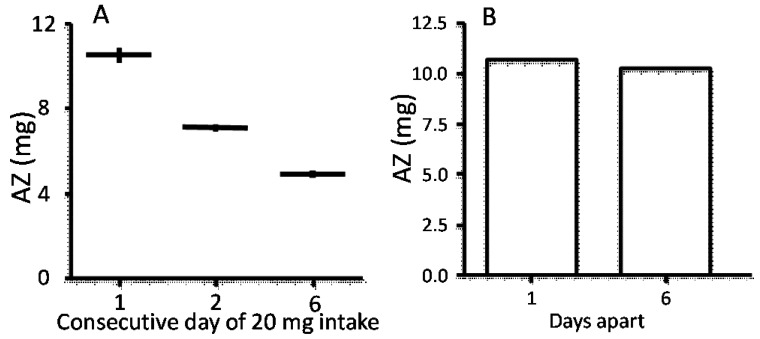
The absorbed zinc (AZ) in adults in the post-absorptive state after ingestion of a 20 mg zinc oral dose (IZ) for 1, 2 and 6 consecutive days (**A**); The AZ in adults in the post-absorptive state after ingestion of a 20 mg zinc oral dose 6 days apart (**B**).

Daily zinc supplementation has been shown to reduce FAZ and subsequently AZ. This is consistent with preliminary data demonstrating that that ingestion of a 20 mg zinc oral dose for 6 consecutive days in adults in the post absorptive state resulted in a 50% reduction in FAZ. This indicates that the efficiency of zinc absorption was halved when 20 mg zinc was given for six consecutive days prior to the measurement of absorption, with nearly as great a reduction when the 20 mg dose was given for only one day before the 20 mg test dose (Figure 2). 

While some effect of down-regulation of one or more transporters involved in the intestinal absorption of zinc cannot be completely excluded, it is likely that zinc transporters on the enterocytes become saturated and have no time to transport all zinc from the previous dose into the portal circulation, thus diminishing the maximal capacity for absorption of the next ingested dose. Such results suggest that maximum absorption and economic advantage is achieved if the supplemental zinc dose is given at 3–5 day intervals. 

### 5.3. The Optimal Zinc Salt?

Five zinc compounds have been listed as generally being recognised as safe by the US Food and Drug Administration. They are zinc sulphate, zinc chloride, zinc gluconate, zinc oxide and zinc stearate. At present, little information is available on the pharmacokinetics of these zinc compounds in humans, and there is no consensus regarding the most appropriate form to use. 

Of the zinc compounds approved for human consumption [128], the preferred choices are zinc oxide or zinc sulphate, the two cheapest forms [129]. Studies comparing the absorption of zinc in these different chemical forms have demonstrated no difference in absorption [130] consistent with other studies of food fortified with either zinc oxide or sulphate [130,131,132,133]. 

### 5.4. Zinc Supplementation and Its Relationship to Markers of Zinc Deficiency

Despite the high prevalence of zinc deficiency in the human population, there are no methods currently available for sensitively and accurately assessing zinc status in individuals. Zinc depletion/repletion studies in both animals and humans have provided some insight into the potential markers of zinc status. Unfortunately, plasma zinc is not sensitive for clinical zinc deficiency; plasma zinc concentration can fall in response to factors including infection, inflammation, stress and trauma. Whereas fasting can release zinc into the circulation, causing an increase in plasma zinc levels [134]. The interpretation of plasma zinc concentration requires knowledge of all of these possible confounders. Studies have investigated the responses of zinc transporter proteins, ZnT1, ZIP8, ZIP10 and potentially other ZnT/Zip genes and proteins, suggesting it have the capability to serve as a biomarker for dietary zinc depletion [72,135,136,137]. There have been great expectations that assays of the activity of selected zinc metalloenzymes would provide invaluable markers of zinc status [138,139]. However, there have been no studies to date to confirm the utility of several enzyme assays to be reliable markers of zinc status. More recently, other novel markers have been postulated to serve as biomarkers for marginal zinc status in humans, including fibrin β [140], specific blood gene transcripts, circulating microRNAs (miRNA), and cytokines [141]. Furthermore, Reed *et al.* [142] suggest that the blood linoleic acid: dihomo-γ-linolenic acid (LA:DGLA) ratio may be a sensitive physiological markers of zinc status. 

It has been shown that with the use of a stable isotope tracer of zinc, the zinc pool that exchanges rapidly with plasma zinc may provide novel information on exchangeable zinc pools in clinical situations [143]. The analysis of zinc pools that exchange rapidly with plasma is important as zinc is required for physiologic functions including maintenance of zinc-dependent functions and must, therefore, be readily available [83]. If this assumption is true, it then becomes important to determine the minimum size of EZP required to support the optimal function of zinc dependent metabolic processes [83,143]. Furthermore, mathematical models of zinc kinetics may assist the continuing search for a marker of zinc status by providing a more detailed understanding of zinc metabolism in various situations, against which novel methods can be evaluated [144]. More extensive research, especially in children at risk of zinc deficiency, is needed however to define the utility of EZP as a zinc status biomarker.

### 5.5. Zinc Supplementation and Its Relationship to Intestinal Disease

Zinc supplementation might be beneficial to gut function. However, in conditions where the intestine is inflamed and/or compromised, such as diarrhoea and environmental enteropathy, the efficacy of zinc may be reduced due to impaired absorption, loss of zinc or a combination of both. There are limited studies using zinc stable isotope techniques in these settings. For example, Manary *et al.* [43] reported that Malawian children at risk for environmental enteropathy and zinc deficiency had perturbed zinc homeostasis and impaired absorption as assessed by EFZ and intestinal permeability, respectively. In addition, Tran *et al.* [58] reported that children with coeliac disease had decreased EZP compared to control subjects. These studies suggest that impaired zinc homeostasis and absorption is associated with a compromised gastrointestinal tract [145]. To be informative, it is crucial that tracer studies be undertaken in such children. 

There are studies utilising zinc stable isotope techniques in children in low-middle income countries, where intestinal inflammatory disease, such as environmental enteropathy, is prevalent due to poor hygiene and living conditions (Table 1). These studies provide useful information on zinc metabolism and homeostasis to assist in providing optimal zinc supplements for maximum benefits. Such studies address the effect of zinc dose [146,147,148], fortification with zinc [132,149] habitual diets [37,43] and avoiding high phytate diets [36,73,150,151,152] However, there are no studies to date applying zinc stable isotope techniques to assess zinc metabolism and homeostasis in children in low-middle income countries with acute diarrhoea. Further, there is only one study on environmental enteropathy [43].

**Table 1:** Summary of studies that utilise the zinc stable dual isotope tracer ratio (DITR) technique to assess fractional absorption of zinc (FAZ), absorbed zinc (AZ), total absorbed zinc (TAZ), endogenous faecal zinc (EFZ) and exchangeable zinc pools (EZP) in children in low-income countries.

Reference	Context	Zinc Intake	Zinc Status Parameters Using DITR Technique			Key Findings
			FAZ/AZ/TAZ	EZP	EFZ	
Ariff *et al.*, 2014 [146]	Pakistan; healthy breastfed infants (6 months)	10 mg/day for 6 month	√	√		↑ AZ, EZP
Esami *et al.*, 2014 [147]	Kenya; healthy breastfed infants (9 months)	5 mg/day for 3 month	√			↔ AZ
Hambidge *et al.*, 2007 [36]	Guatemala; healthy children (8.9 ±1.3 years)	low-, isohybrid- and control-phyate maize			√	↔ EFZ
Herman *et al.*, 2002 [132]	Indonesia; healthy children (4–8 years)	fortified flour meal 60 mg Zn/kg (as ZnO or ZnSO_4_)	√			↔ AZ between ZnO or ZnSO_4_
Hettiarachchi *et al.*, 2004 [149]	Sri Lanka; healthy children (7–10 years)	fortified rice flour 60 mg/kg (ZnO) for 2 weeks	√			addition of Na_2_EDTA improve zinc absorption
Hettiarachchi *et al.*, 2010 [153]	Sri Lanka; healthy children (4–7 years)	meal 1.5 mg Zn (ZnSO_4_)	√			↔ AZ
Islam *et al.*, 2013 [154]	Bangladesh; healthy non breastfed children (36–59 months)	high-zinc rice (HZnR), conventional rice (CR), or CR+zinc for 1 day	√			↔ TAZ between CR and HZnR ↑ TAZ for CR+Zn
Kennedy *et al.*, 2010 [150]	Malawi; healthy children (2–5 years)	maize high-phytate or maize reduced-phytate diets for 40 days			√	↔ EZP
Kodkany *et al.*, 2013 [155]	India; healthy children (22–35 months)	zinc-rich dry pearl millet flour for 1 day	√			zinc biofortified pearl millet adequately meet the physiological requirements
Li *et al.*, 2015 [156]	China; healthy children (13 ± 1.1 years)	3 mg Zn for 10 days +NaFeEDTA-fortified soy sauce	√			↔ FAZ
Lopez de Romana *et al.*, 2005 [66]	Peru; children at risk of zinc deficiency (3–4 years)	0, 3, 9 mg Zn/100 g flour for 7 weeks	√			↔ AZ before and after
Manary *et al.*, 2010 [43]	Malawi; children with tropical enteropathy (3–5 years)	habitual diet	√		√	↑ EFZ compared to healthy
Manary *et al.*, 2002 [73]	Malawi; healthy children (2–5 years)	maize-based diet + 1.5–2 mg Zn for 1 day	√		√	↑ EFZ compared to previous studies
Manary *et al.*, 2000 [151]	Malawi; children hospitalised for tuberculosis (3–13 years)	corn+soy porridge (low or high phytate) for 3–7 days	√		√	low phytate ↑ FAZ, TAZ
Mazariegos *et al.*, 2006 [152]	Guatemala; healthy children (6–11 years)	low-phytate, isohybrid wild-type or a local maize for 10 weeks	√			↔ FAZ, TAZ
Nair *et al.*, 2013 [157]	India; healthy adolescent (13–15 years)	standardized rice meal or the same meal with 100 g of guava fruit (2.7 mg Zn) for 2 days	√			↔ FAZ
Sheng *et al.*, 2006 [37]	China; healthy children (19–25 months)	habitual diet	√		√	mean intake and absorption of zinc are low compared to average dietary requirements
Zlotkin *et al.*, 2006 [148]	Ghana; healthy children (12–24 months)	5 or 10 mg Zn for 14 days	√			↑ TAZ for high zinc

## 6. Summary and Conclusions

We lack certain necessary pieces of information about zinc to optimise clinical interventions to suit the context, whether as a nutritional supplement or a therapeutic (e.g., for acute diarrhoea). That which can be provided by stable isotope studies includes:

- Data demonstrating the maximal plasma/serum zinc concentration obtained relative to dose (which relates to optimal absorption of zinc using zinc stable isotope kinetics), duration of effect and timing of the doses. These data can be provided using zinc stable isotope techniques [158,159,160,161].
- Delivery of zinc to the target tissue as informed by the volume of distribution, by plasma protein binding and, if needed, tissue biopsy assays. Zinc stable isotope studies have provided limited data [162,163] especially from EZP but more research in this area is warranted.
- Proof of concept demonstrating relationship of a clinical response to a pharmacodynamics or pharmacokinetic marker. At this stage, there has been very little work relating stable isotope measurements such as FAZ, TAZ, EZP and so on to zinc administration and clinical benefit.

More research that focuses on the application of zinc stable isotope techniques to assess zinc kinetics in relation to zinc salt, dose/intake and to physiologic and pathologic conditions is needed. Understanding what dose, frequency and duration will maximize the acceptance and benefits of zinc supplementation whether given as a nutritional supplement to prevent illness related to zinc deficiency or to treat childhood diarrhoea is not possible with the currently available information. The application of zinc stable isotopes tracer techniques provides the opportunity to define the optimal zinc dosing regimen (dose, frequency, duration) relative to amount absorbed and replenishment of the EZP. These effects in turn can be related to health benefit in staged studies. Compartmental modelling will allow the determination of zinc pool sizes and rate constants and fluxes between these pools [39], which can be achieved through simple algebraic equations for data processing [41]. Moreover, the use of zinc stable isotope techniques with saturation response and compartmental modelling will provide a fundamental tool to advance our understanding of zinc absorption, metabolism and homeostasis. 
